# Patient-Reported Outcome Results From the Open-Label, Randomized Phase III Myeloma X Trial Evaluating Salvage Autologous Stem-Cell Transplantation in Relapsed Multiple Myeloma

**DOI:** 10.1200/JCO.18.01006

**Published:** 2019-04-10

**Authors:** Sam H. Ahmedzai, John A. Snowden, Andrew John Ashcroft, David Allan Cairns, Cathy Williams, Anna Hockaday, Jamie D. Cavenagh, Debo Ademokun, Eleni Tholouli, David Allotey, Vijay Dhanapal, Matthew Jenner, Kwee Yong, Jim Cavet, Hannah Hunter, Jennifer M. Bird, Guy Pratt, Christopher Parrish, Julia M. Brown, Treen C.M. Morris, Gordon Cook, on behalf of the National Cancer Research Institute Haemato-Oncology Clinical Studies Group

**Affiliations:** ^1^ The University of Sheffield, Sheffield, United Kingdom; ^2^ Sheffield Teaching Hospitals NHS Foundation Trust, Sheffield, United Kingdom; ^3^ Pinderfields Hospital, Mid-Yorks NHS Trust, Wakefield, United Kingdom; ^4^ University of Leeds, Leeds, United Kingdom; ^5^ Nottingham City Hospitals, Nottingham City, United Kingdom; ^6^ Barts Health NHS Trust and The London NHS Trust, London, United Kingdom; ^7^ Ipswich Hospital NHS Trust, Ipswich, United Kingdom; ^8^ Manchester Royal Infirmary, Manchester, United Kingdom; ^9^ Royal Derby Hospital, Derby, United Kingdom; ^10^ Medway Maritime Hospital, Gillingham, United Kingdom; ^11^ University Hospital Southampton NHS Foundation Trust, Southampton, United Kingdom; ^12^ University College Hospital, London, United Kingdom; ^13^ The Christie NHS Foundation Trust, Manchester, United Kingdom; ^14^ Plymouth Hospitals Trust, Plymouth, United Kingdom; ^15^ University Hospitals Bristol NHS Trust, Bristol, United Kingdom; ^16^ University Hospitals Birmingham NHS Foundation Trust, Birmingham, United Kingdom; ^17^ Queen’s University, Belfast, United Kingdom

## Abstract

**PURPOSE:**

Salvage autologous stem-cell transplantation (sASCT) in patients with multiple myeloma (MM) relapsing after a prior autologous stem-cell transplantation leads to increased remission duration and overall survival. We report a comprehensive study on patient-reported outcomes, including quality of life (QoL) and pain in sASCT.

**METHODS:**

Patients were randomly assigned to either sASCT or nontransplantation consolidation (NTC). Pain and QoL were assessed as secondary outcomes using validated QoL instruments (European Organisation for Research and Treatment of Cancer QLQ-C30 and myeloma-specific module, QLQ-MY20; the Brief Pain Inventory [Short Form]; and the Leeds Assessment of Neuropathic Symptoms and Signs [Self-Assessment] scale).

**RESULTS:**

A total of 288 patients (> 96%) consented to the QoL substudy. The median follow-up was 52 months. The European Organisation for Research and Treatment of Cancer QLQ-C30 Global health status scores were higher (better) in the NTC group at 100 days after random assignment (*P* = .0496), but not at later time points. Pain interference was higher (worse) in the sASCT group than in the NTC group at 6 months after random assignment (*P* = .0267), with patients with sASCT reporting higher scores for Pain interference with daily living for up to 2 years after random assignment. Patients reporting lower concerns about adverse effects of treatment after sASCT had a time to progression advantage.

**CONCLUSION:**

Patients with sASCT with relapsed MM demonstrated a comparative reduction in QoL and greater impact of treatment adverse effects lasting for 6 months and up to 2 years for pain, after which patients who had received sASCT reported better outcomes. Patients who experienced lower adverse effects after sASCT had longer time to progression and overall survival, showing the need to improve symptom management peritransplantation. To our knowledge, this study provides the most comprehensive picture of QoL before and after sASCT in patients with relapsed MM.

## INTRODUCTION

Despite advances in novel antimyeloma agents,^1-4^ the use of a salvage autologous stem-cell transplantation (sASCT) has continued to represent an option in well-selected patients with multiple myeloma (MM) because of the potential for sustained disease control. sASCT was originally supported by retrospective, registry-based, or single-center analyses,^5-8^ but definitive evidence of its efficacy in terms of significantly improved time to progression (TTP), progression-free survival (PFS), and overall survival (OS) was provided by the UK National Cancer Research Institute Myeloma X trial.^9,10^

With improving survival, MM is being experienced by an increasing proportion of patients as a chronic illness.^11,12^ However, even myeloma in remission may be associated with many symptoms, which often arise as a consequence of myeloma itself, its treatment, and interactions with comorbid conditions accompanying aging. A cumulative burden of symptoms and treatment adverse effects, including various forms of pain, all affect health-related quality of life (QoL).^13,14^ There has been growing interest in the methodology of evaluating patient-reported outcomes (PROs) to measure the impact of both novel agents and autologous stem-cell transplantation (ASCT) on QoL in patients with MM.^15-18^ Studies have also reported on PROs in larger cohorts of patients undergoing ASCT and allogeneic transplantations for other conditions and in older populations.^19-23^ However, few studies have examined the years after transplantation that patients with MM can now expect to survive.

A secondary aim of Myeloma X was to evaluate the impact of sASCT compared with nontransplantation consolidation (NTC) with oral cyclophosphamide once per week on PROs relating to QoL and pain at first relapse after a prior ASCT and after reinduction chemotherapy. The hypothesis was that ASCT was expected to be superior to NTC in terms of TTP. Therefore, ASCT-related toxicity in the short term and its potential impact on QoL, as well as patients’ long-term QoL, were of interest. Furthermore, we sought to evaluate the association of QoL at random assignment with subsequent clinical outcomes and to identify patient subgroups that may gain most QoL benefit from sASCT.

## METHODS

### Study Design

**FIG 1.**
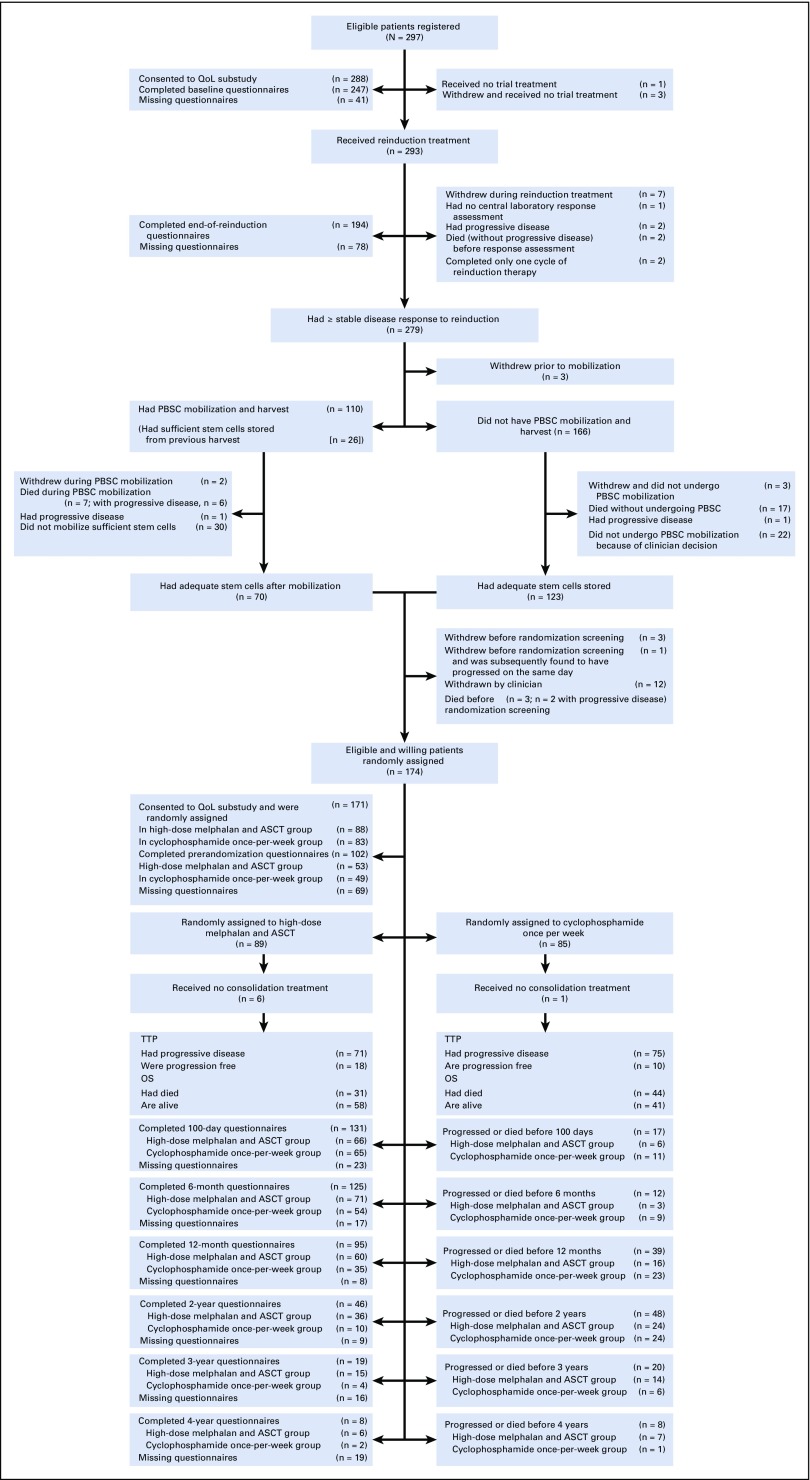
CONSORT diagram for Myeloma X study, including details of European Organisation for Research and Treatment of Cancer (EROTC) QLQ-C30 and MY20 questionnaire return. ASCT, autologous stem-cell transplantation; OS, overall survival; PBSC, peripheral-blood stem cell; QoL, quality of life; TTP, time to progression.

Patients with symptomatic, measurable MM were eligible if they required treatment of first progressive disease (as defined by the International Myeloma Working Group criteria^24^ at least 18 months after a prior ASCT). Inclusion and exclusion criteria and the trial procedures have been described in detail previously^9,10^ and are summarized in the trial CONSORT diagram (Fig 1) and Data Supplement.

### End Points

The primary end point was TTP; secondary end points were response rate, PFS, OS, toxicity and safety, pain, and QoL.

### Patient-Reported Outcomes

Pain and QoL were assessed in patients consenting to the QoL substudy. These patients were willing and able to complete the pain and QoL questionnaires at specified time points throughout the study. All instruments were self-administered to avoid interviewer bias and to enable the patients to complete the questionnaires at home if they preferred.

The European Organization for Research and Treatment of Cancer Quality of Life Questionnaire (EORTC QLQ-C30) and the EORTC myeloma-specific module (EORTC QLQ-MY20), were used to assess QoL during reinduction and randomized treatment (Data Supplement). Pain experience was captured using the Brief Pain Inventory (Short Form) [BPI-SF], which assesses the severity of pain and its impact on aspects of daily living in patients with chronic pain.^25^ The Leeds Assessment of Neuropathic Symptoms and Signs (Self-Assessment; S-LANSS) pain scale was administered as a diagnostic tool to assess the extent to which the pain was neuropathic.^26^

Questionnaires were administered before registration and after completion of reinduction. For randomly assigned patients, the questionnaires were administered before randomization, unless within 2 weeks of the previous questionnaires. These questionnaires were administered in the clinic. Questionnaires were then sent by mail at 100 days after random assignment, 6 and 12 months after random assignment, and then annually until the last randomly assigned patient was 2 years after random assignment (Fig 1) in the absence of disease progression. In this analysis, all data up to 2 years after random assignment are included.

### Statistical Analysis

Sample size and trial details have been described previously.^9,10^ The cutoff date for this analysis was July 14, 2015. The intention-to-treat population for the QoL substudy included all consenting patients who had completed at least one questionnaire. All statistical analyses were undertaken in SAS (version 9.4; SAS Institute, Cary, NC). All statistical tests were two-sided and were considered significant at the 5% level.

sASCT was expected to be superior to NTC in terms of TTP. Therefore, ASCT-related toxicity in the short term and its potential impact on QoL, as well as patients’ long-term QoL, were of interest. EORTC QLQ-C30, EORTC QLQ-MY20, the S-LANSS pain scale, and the BPI-SF were used to measure temporal changes in patient-assessed QoL and pain, and their association with treatment. Of individual interest were the QLQ-C30 Global health status score, BPI-SF Pain interference scale, and MY20 Side effects of treatment subscale. Adjustments to the 5% significance level were not made for repeating the analysis for these scales because they were defined prospectively as being of individual interest in the study Statistical Analysis Plan, which was finalized before any analysis was undertaken. No power calculations were prespecified for these prospectively defined scales. For all other QLQ-C30, QLQ-MY20, BPI-SF subscales, and the S-LANSS, no statistical hypothesis testing was performed to compare between treatment groups.

QoL subscales were summarized using means, 95% CIs, and differences between the two groups. For randomly assigned patients, multilevel repeated mixed model analyses, which allowed for randomized treatment, time effects, treatment-time interactions, and baseline QoL (fixed effects), and patient and patient-time interaction (random effects) were used. Additional details are given in the Data Supplement.

## RESULTS

### Patients and Treatment

Between April 16, 2008, and November 19, 2012, 288 of the 297 patients registered consented to the QoL substudy; 174 patients were subsequently randomly assigned to receive sASCT (n = 89; 88 consented to the QoL substudy) or NTC (n = 85; 83 consented). Baseline demographic and disease characteristics were well balanced between the treatment groups (Table 1), except for a higher proportion of patients with International Staging System III in the transplantation group. Patients consenting to the QoL substudy were similar to those who did not consent at registration and randomization (Data Supplement).

**TABLE 1.**
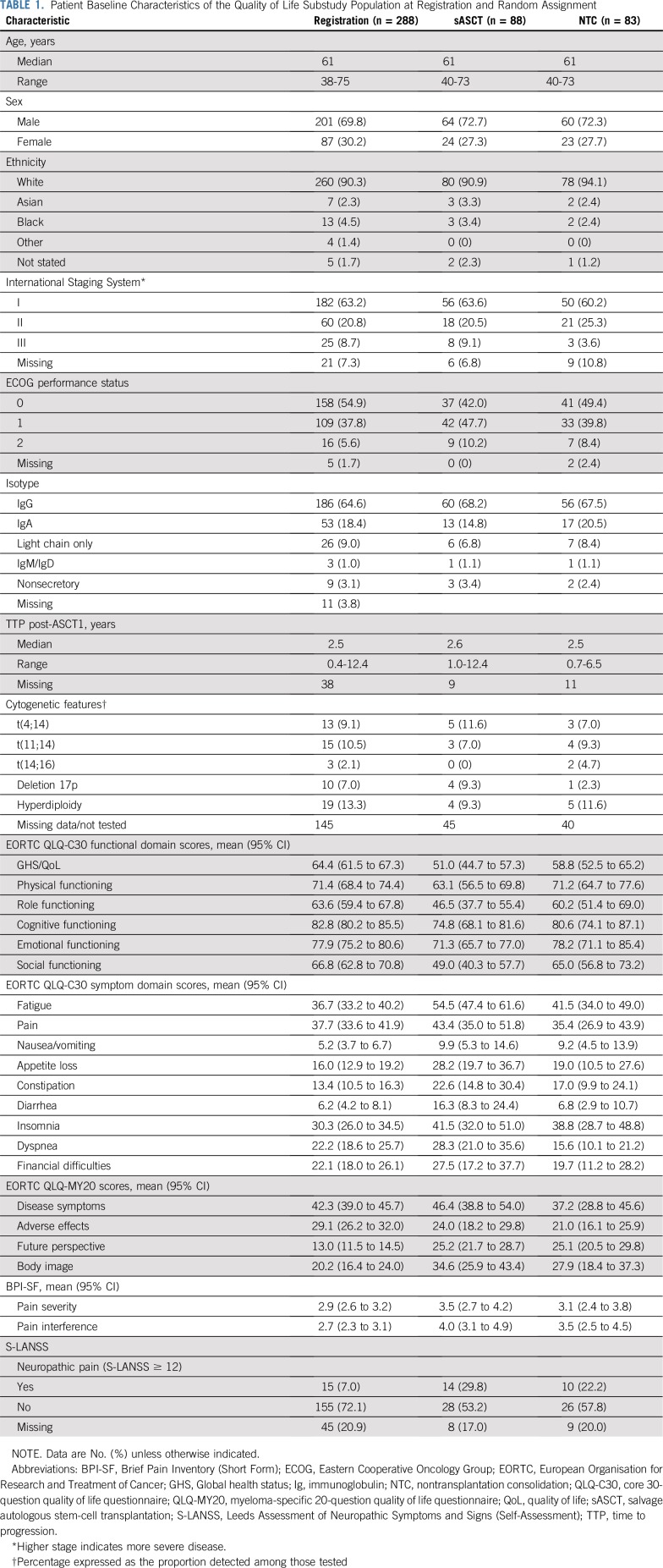
Patient Baseline Characteristics of the Quality of Life Substudy Population at Registration and Random Assignment

### Questionnaire Compliance

Compliance was good, with 76.1% of all expected questionnaires returned among consenting patients and similar rates of compliance comparing randomly assigned groups (Data Supplement). The greatest rate of noncompliance for EORTC QLQ-C30 and QLQ-MY20 questionnaires was at the randomization baseline time point (59.6% complying; Data Supplement). For the S-LANSS and BPI-SF questionnaires, compliance was slightly less (54.4% and 53.8%, respectively; Data Supplement).

Subsequently, among the randomly assigned QoL population, more patients in the sASCT group completed the QLQ-C30, MY20, and other questionnaires at later time points, owing to the increased TTP (median, 19 months; 95% CI, 16 to 26 months; *v* 11 months; 95% CI, 9 to 12 months]; hazard ratio [HR], 0.45; 95% CI, 0.31 to 0.64; log-rank *P* < .001; Data Supplement).

### QoL and Pain With Respect to Reinduction Treatment

The adjusted means and 95% CIs (Data Supplement) with baseline mean scores (Table 1) show that the majority of adjusted subscale means had changed compared with the trial entry subscale means. Global health status, Fatigue, and Diarrhea demonstrated medium-sized, clinically relevant differences.^27^ Role functioning, Social functioning, Nausea/vomiting, Appetite loss, Constipation, Insomnia, and Dyspnea demonstrated small clinically relevant differences.^27^ The BPI-SF Pain severity score was similar to trial entry, but the Pain interference score increased slightly (Data Supplement).

### QoL and Pain With Respect to Randomized Treatment

**FIG 2.**
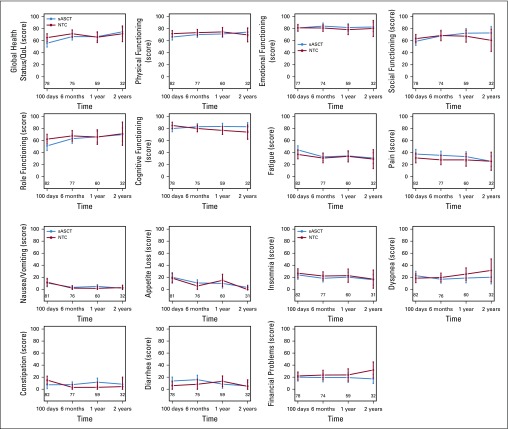
Baseline adjusted complete-case summary statistics (mean and 95% CI) scales from the European Organisation for Research and Treatment of Cancer (EROTC) QLQ-C30 questionnaire in randomly assigned patients. Global health status/quality of life (QoL)-social functioning: higher score represents better QoL/functioning; fatigue-body image loss: higher score represents worse symptoms/QoL. NTC, nontransplantation consolidation; sASCT, salvage autologous stem-cell transplantation.

**FIG 3.**
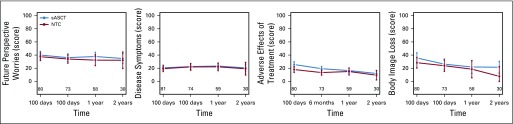
Baseline adjusted complete-case summary statistics (mean and 95% CI) scales from the European Organisation for Research and Treatment of Cancer (EORTC)-QLQ-MY20 questionnaire in randomly assigned patients. Future perspective worries-body image loss: higher score represents worse symptoms/quality of life (QOL). NTC, nontransplantation consolidation; sASCT, salvage autologous stem cell transplantation.

Figures 2 and 3 and the Data Supplement show the adjusted mean subscale scores of the EORTC QLQ-C30 and MY20 and the mean BPI-SF scores. It should be noted that there were imbalances at randomization baseline in a number of subscales, including Global health status (Table 1). In the majority of patients, the QoL subscale seemed to be worse in the sASCT group. The reasons for this imbalance could not be identified.

Global health status subscale was significantly different between groups at 100 days after random assignment (*P* = .0496; Table 2), but at no other time points after random assignment (Table 2). The Global health status score was higher at 100 days after random assignment by 9.2 points in the NTC group of the trial—a small-medium size difference.^27^ This deterioration in Global health status for patients receiving sASCT compared with NTC dissipated to a trivial^27^ difference at 6 months and a smaller trivial difference at 1 year. The difference at 2 years favored sASCT but was still trivial. The Side effects of treatment subscale was higher in the sASCT group of the trial at 100 days and 6 months after random assignment (Table 2; Fig 2). This difference was small (7.6 and 5.9 points at 100 days and 6 months, respectively) and subsequently dissipated.

**TABLE 2.**
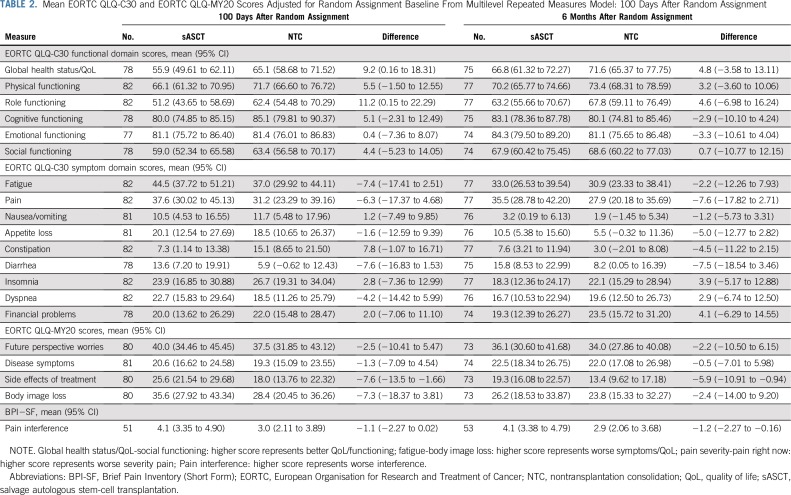
Mean EORTC QLQ-C30 and EORTC QLQ-MY20 Scores Adjusted for Random Assignment Baseline From Multilevel Repeated Measures Model: 100 Days After Random Assignment

There was no significant difference between the trial groups for Pain interference, adjusted for baseline score and baseline neuropathic pain level at the 100-day postrandomization time point (*P* = .0602; Table 2); there were significant differences at 6 months (*P* = .0267; Table 2) and similar significant differences up to 2 years (Table 3; Data Supplement). In all of the time points considered, Pain interference was approximately 1 point lower in the NTC group, a clinically relevant difference. Sensitivity analysis under an assumption of data missing at random using multiple imputation by chained equations provided similar estimates, but in the case of Global health status, this was not significant (Data Supplement).

**TABLE 3.**
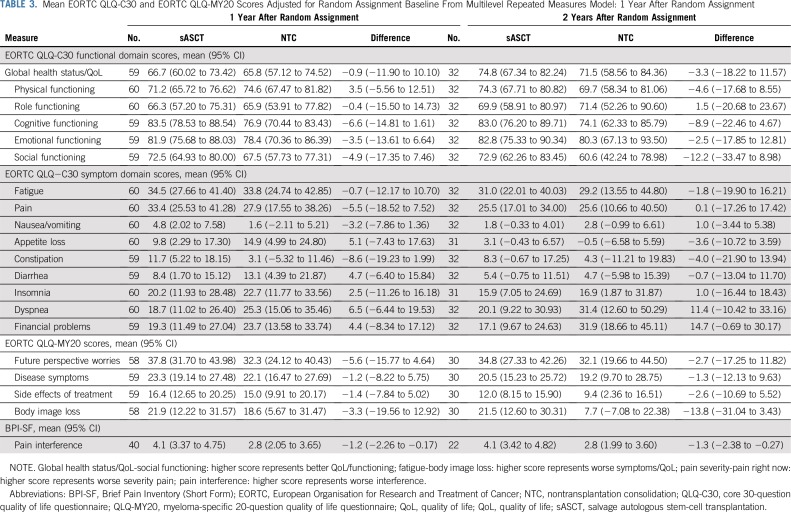
Mean EORTC QLQ-C30 and EORTC QLQ-MY20 Scores Adjusted for Random Assignment Baseline From Multilevel Repeated Measures Model: 1 Year After Random Assignment

### Subgroup Analysis

Post hoc exploratory analysis of key patient subgroups that had previously been investigated for clinical outcomes (TTP and OS) showed no significant heterogeneity of treatment effect on subscales of special interest: Global health status (Data Supplement), Side effects of treatment (Data Supplement), and BPI-SF Pain interference score (Data Supplement). The greatest variability in estimates was observed in the adverse cytogenetics risk group, which was small for the intersection of patients with completed questionnaires and available cytogenetics.

### Association Between Baseline Scales and Outcome

Post hoc exploratory analysis of TTP by randomly assigned allocation suggested that patients with Global health status greater than median at randomization (Data Supplement) and who received sASCT had a significant TTP advantage over those receiving NTC (HR, 0.3; 95% CI, 0.15 to 0.61; *P* = .006). However, with multivariable Cox regression analysis accounting for stratification factors, this difference was not significant. Patients who reported a lower than median score on the Side effects of treatment subscale randomization and who received sASCT had a significant TTP advantage over those receiving NTC (HR, 0.24; 95% CI, 0.10 to 0.55; *P* = .003). This survival difference was maintained with multivariable Cox regression analysis (HR, 0.20; 95% CI, 0.04 to 1.00; *P* = .0499). Pain scores were not found to be strongly predictive of outcome (TTP and OS; Data Supplement).

## DISCUSSION

Bony pain is a significant feature of myeloma and persists even when the disease is controlled because of both a lack of bone repair and the mechanical stresses of movement. This can persist into remission when the disease is no longer active and after systemic anticancer treatments have ceased.^11,28,29^ This trial provides the most comprehensive picture of PROs before, immediately after, and for a substantial time after sASCT.

Several studies have sought to describe the process of MM treatment, including adverse effects of ASCT in terms of PROs and overall QoL. Morbidity during ASCT is high, with mucositis, emesis, and complications of bone marrow depletion and sepsis. Many patients find this period acutely distressing and may display long-term psychological changes. Sherman et al^14^ assessed a sample of 94 patients undergoing ASCT using the Functional Assessment of Cancer Therapy–Bone Marrow Transplant (FACT-BMT) and symptom scales. They found that levels of physical and functioning well-being were already low, and fatigue, anxiety, and depression pain were prevalent at the pretransplantation assessment. After ASCT, there was a worsening of transplantation-related concerns, depression, and life satisfaction. Pain and physical functioning did not deteriorate. Older patients were no more compromised than younger ones, confirmed in another study of mixed hematologic malignancies.^15^

Jim et al^23^ described the QoL effects of ASCT in MM in a sample of 701 participants in the Blood and Bone Marrow Transplantation Clinical Trials Network 0902 trial. Using the Medical Outcomes Study Short Form (a generic instrument describing key dimensions of daily life), they were able to describe four trajectories for physical and mental aspects of QoL 100 days and 6 months after ASCT: low and stable; average and declining, then stable; higher than average and stable; and average and stable. They also concluded that attrition in post-ASCT studies could give rise to overestimates of QoL, but these are slight and consistent over time.

Novel agents in triplet combinations and their impact on PROs have recently been reported in relapsed patients for carfilzomib,^30^ ixazomib,^31^ and elotuzumab,^32^ where 43% to 62% of patients in these studies were treated for first relapse. Baseline and long-term on-treatment scores for the scales of key interest were similar to those observed in this study. Each novel agent improved PFS and showed no detrimental long-term impact on Global health status. These findings are important because others have shown that baseline psychosocial factors and QoL after induction and before ASCT, and the extent of treatment adverse effects during ASCT, could be predictive of post-transplantation adverse changes, including the finding of post-traumatic stress disorder in some patients.^33-35^ Most recently, O’Sullivan et al^36^ confirmed that higher pretransplantation pain predicted lower physical well-being and QoL. However, it should be noted that these studies included a variety of hematologic malignancies, both autograft and allograft, and a range of QoL measures.

Although the questionnaire compliance was good, a limitation of this report is the low compliance at randomization baseline for all questionnaires, which leads to a subsample of data used in statistical models. In addition, there was an imbalance in the QoL scores at randomization baseline, with most scores better in the cyclophosphamide once-per-week group. There was no reason identified for the reduced compliance nor the baseline imbalance, with no significant differences identified in the time between trial registration, randomization, and questionnaire completion, or an imbalance in the same subsample of patients at trial registration or the end of reinduction. However, sensitivity analysis found no evidence to reject an assumption of missing at random, and multiple imputation provided broadly similar results, with potentially a nonsignificant difference in Global health status at 100 days post-ASCT.

We found that after entering the trial and after reinduction with bortezomib, doxorubicin, and dexamethasone, there were adverse changes in many aspects of QoL reflecting the intensity of treatment; these were of medium clinical significance only for Global health status, Fatigue, and Diarrhea. Importantly, psychological aspects were not compromised, because these have been shown to predict worse outcomes after hematopoietic stem-cell transplantation (HSCT) elsewhere.^33^ In the early postrandomization period, patients who had undergone sASCT showed evidence of a small reduction in aspects of QoL. At 100 days, only Role functioning and Global health status were significantly higher in the NTC arm, with a magnitude indicating a small clinical difference. However, by 1 year after random assignment, this difference in Global health status was not observed.

There was evidence from the S-LANSS scale that the neuropathic component of pain was increased at 100 days after transplantation, but not later. Pain interference with daily living using the BPI-SF also increased in patients with sASCT and was seen until 2 years after random assignment. Drugs used in the ASCT process are not usually associated with direct neuropathic changes. However, systemic inflammation can give rise to increased pain sensitivity, probably because of the effect of proinflammatory cytokines directly sensitizing peripheral pain pathways.^37^ We have shown in another cohort study of patients with MM in remission that pain level as measured by BPI-SF was correlated with circulating interleukin-6.^38^ In sASCT, the increased risk of systemic inflammation, including sepsis, could condition patients with existing neural damage to express more pain. Prolonged increased pain after HSCT has not been reported previously, possibly because others have not used a standardized pain measure such as BPI-SF, with its more sensitive, specific Pain interference scale.

The MY-20 module has a specific scale for detecting concerns about Side effects of treatment. Although this score was increased in patients randomly assigned to sASCT at 100 days and 6 months, the magnitude of this change was small and disappeared subsequently. Patients with sASCT who reported a lower (ie better) than median level of concern about adverse effects had significant TTP and OS advantages, even after adjustment in a multivariable Cox regression analysis. One likely interpretation of this in patients undergoing ASCT is that maximal efforts should be made to minimize the emergence of adverse effects of the procedure and to optimize their management. El-Jawahri et al^39^ reported on the effect of a randomized trial of in-patient palliative care on QoL of patients with HSCT. This novel intervention led to a smaller increase in depression, lower anxiety, and less increase in symptom burden at 2 weeks post-HSCT and also a smaller reduction in QoL at 2 weeks, with higher QoL at 3 months.

This study is subject to several limitations, including an open-label design, in which lack of blinding could be relevant where subjective end points such as pain, other symptoms, treatment adverse effects, and QoL are of interest. We relied on patient reports using paper-based questionnaires. These are subject to problems related to adherence, especially when patients are experiencing periods of increased illness. We found the adherence and missing data rates to be comparable to other studies in this population, using the same or similar instruments. Attrition rates are possibly a source of bias if they occur differentially in allocated treatment arms. It has been shown that attrition can overestimate QoL scores, but this effect can be consistent over time and between allocated treatment arms.^23^ This is perhaps relevant in this study, where PROs were collected up until the time of disease progression.

The small and diminishing differences in Global health status and Side effects of treatment need to be considered alongside the results of Myeloma X, which showed a significant benefit of sASCT on OS. The benefits of sASCT should be considered alongside the relatively short-term negative effects on QoL and pain when making patient treatment decisions and further support the use of sASCT.
